# Joint associations of smartphone use and gender on multidimensional cognitive health among community-dwelling older adults: a cross-sectional study

**DOI:** 10.1186/s12877-019-1151-x

**Published:** 2019-05-24

**Authors:** Manqiong Yuan, Jia Chen, Zi Zhou, Jiahui Yin, Jielong Wu, Mingliang Luo, Lixia Wang, Ya Fang

**Affiliations:** 1 State Key Laboratory of Molecular Vaccinology and Molecular Diagnostics, School of Public Health, Xiamen University, Xiang’an Nan Road, Xiang’an District, Xiamen, 361102 Fujian China; 2 Key Laboratory of Health Technology Assessment of Fujian Province University, School of Public Health, Xiamen University, Xiang’an Nan Road, Xiang’an District, Xiamen, 361102 Fujian China

**Keywords:** Multi-domain cognitive health, Smartphone use, Proportional-odds cumulative logit model, Joint association, Older adults

## Abstract

**Background:**

Smartphone use has become an increasingly pervasive part of our daily lives, and as a portable media device, smartphones provide good support for cognitive training during aging. However, little is known about the joint association of smartphone use and gender on the cognitive health of older adults, particularly with regard to multi-domain cognition.

**Methods:**

A face-to-face survey of 3230 older adults aged 60+ years was conducted in Xiamen, China, in 2016. The Montreal Cognitive Assessment (MoCA) score was used to measure both general and multi-domain cognition. Smartphone use was self-reported and the number of the smartphone functions used (NSFU) was classified as 0, 1, and 2+. General and subdomain cognitive functions were modelled on NSFU only, gender only, and NSFU and gender combined by using a series of proportional-odds cumulative logit models. Furthermore, joint associations of gender and NSFU on both general and multi-domain cognition were estimated, and a four-category quantile classification was used to evaluate the total MoCA score.

**Results:**

Among all 3230 respondents, 2600 remained after exclusion of respondents with very low MoCA scores (below the education-adjusted cut-offs for dementia). Only 29.96% of older adults used smartphones, 473 (60.72%) of which were men. Respondents who had a higher NSFU maintained a better general and sub-domain cognition except for memory and orientation. Although women had lower values compared to men in visuospatial ability (OR (95% CI): 0.46 (0.37–0.57)), they outperformed their male counterparts in memory (OR (95% CI): 1.38 (1.10–1.73)). The results of the joint association showed that women’s inferiority in visuospatial ability diminished when they had a NSFU of 2+. However, a significantly better improvement in memory for male was achieved when they had a NSFU of 1 rather than 2 + .

**Conclusions:**

A higher NSFU was positively associated with increased general and partial subdomain cognitive functions. However, gender differences were found in visuospatial ability and memory, which could be alleviated by smartphone use.

## Background

Cognitive decline is one of the most critical public health issues for older adults, as it greatly decreases the independence of affected adults as well as their quality of life. Moreover, cognitive decline imposes a huge emotional and financial burden on their families and society [1]. Older adults with cognitive impairments are experiencing a wide range of difficulties in their daily lives, such as memory loss, confusion of time or place, and challenges with problem solving [2]. According to the World Alzheimer Report 2015, 46.8 million people live with dementia globally, and this number is expected to reach 74.7 million in 2030 and 131.5 million in 2050. By 2015, the global costs of dementia reached 818 billion dollars with an increase of 35.4% from 2010 [3]. In addition, older adults with severe cognitive impairment typically require intensive care and their caregivers also reported to have experienced more stress than other caregivers [4]. A report released by the Institute of Medicine indicated that cognitive health among older adults can be maintained or even improved [5]. Studies of brain tissue in both humans and in animal models showed that neurons do not die but rather, their synaptic structure and function diminish in response to aging [6–9], indicating the possibility for improving cognitive health.

A host of studies also suggested that engagement in cognitive challenging activities, such as watching television, reading, listening to music, or using a computer is related to the maintenance and improvement of cognitive functions in the elderly [10–13]. The smartphone, which is the most widely-used and portable device of the current digital age, serves as an all-in-one device, offering an increasing number of useful and interesting applications for its users involving traditional functions such as phone, messaging, multimedia player, and Internet browser, as well as novel applications (apps) such as social networking and health related apps, to name a few. However, the influence of smartphone use has received little research attention, despite providing a wide range of cognitive challenging activities and being an important cognitive training tool for older adults.

Cognition is multidimensional and encompasses processes related to attention, memory, executive and visuospatial function, and language. Prior studies suggested that performing different technological tasks involves the use of different cognitive domains. For example, an Internet search is related to memory (remembering the appropriate procedure to launch a browser), visuospatial abilities, attention (finding and focusing on relevant information), and executive functions (structuring necessary actions in the correct order) [14]. Furthermore, making a phone call and using social networking are related to the language capability [15]. Positive effects of computer use were reported for selective attention and memory among older adults in a six year follow-up study [16]. Similarly, improved language and memory domains were reported among older participants after participating in 15,120-min lessons on computer learning by Tiago et al. [17]. All of these findings indicate potential benefits of smartphone use on multi-domain cognitive health, due to the versatility and similarity between computer and smartphone.

Abundant evidence indicates that cognitive ability varies with gender. For example, gender differences in the neuropsychological processes involved in spatial-cognition tasks have been demonstrated in prior studies [18, 19], and men have been reported to outperform women in visual–spatial tasks [20, 21]. However, the playing of an action video game can reduce this gender disparity in spatial function [22], indicating that smartphone use may potentially bridge the gender gap in selected cognitive domains. Moreover, gender differences also have been reported in m-learning acceptance. For example, Kimbrough et al. reported that women preferred and more frequently used social media and online video calls than men [23]. This means that sex might influence the usage of smartphone applications, and correspondingly impact different domains of cognition. However, the joint associations of smartphone use and gender on multi-domain cognitive health have not been explored to date.

Therefore, this study investigated the gender differences in the use of smartphone functions and in cognitive ability, as well as the associations between smartphone use and general and, especially, multi-domain cognitive health. Furthermore, this study explored the joint associations between gender and smartphone use on multi-domain cognition. The underlying hypothesis is that smartphone use is positively associated with multi-domain cognition and that the associations between smartphone use and different cognitive domains varied by gender.

## Methods

### Study population

A face-to-face survey was conducted among registered residents aged over 60 years from July 1st to October 20th, 2016, in Xiamen, China. A multistage random sampling method was used for the sampling. The primary sampling unit was the district in Xiamen (involving two urban and four rural districts in total), the secondary sampling unit was the sub-district, and the community in each sub-district was then regarded as the tertiary sampling unit. According to the proportion of eligible older adults in each community, individuals were randomly chosen after controlling for gender and age. Ultimately, a total of 3230 respondents from 44 communities in six districts were interviewed, 3061 (94.77%) of which provided valid responses. A more detailed study design and methods have been reported previously [24]. This study was approved by the ethical review committee of the School of Public Health, Xiamen University. Written informed consent was obtained from each respondent prior to the participation in the questionnaire survey.

### Measures

The primary outcomes of the present study were general and multi-domain cognitive functions, which were evaluated by the Beijing version of the Montreal Cognitive Assessment (MoCA), a Chinese adaptation of the MoCA tailored to the local context. This adaptation has been reported to have good sensitivity as well as specificity for cognitive screening among Chinese elderly [25]. Six cognitive subdomains were extracted from the MoCA [26], including delayed memory (five points), visuospatial ability (four points), executive ability (four points), attention (six points), language (six points), and orientation (six points). Of note, two points were added for older adults with ≤6 years of education, while one point was added for those with 7–12 years of education (if the adjusted score exceeded 30, 30 was assumed for analyses) to overcome the educational bias of the MoCA [27].

The exposure of interest was smartphone usage, assessed by the question of “Do you use a smartphone in your daily life?”, and the two options of yes and no were provided. If the answer was yes, the respondents would be further asked “Which of the following smartphone functions did you use (check all that apply)?” offering a list of 10 items (phone call/message, Internet searching/news reading, social networking services, listening to music or radio, online shopping, video viewing, camera use, playing games, learning, and others). The number of smartphone functions used (NSFU) for each individual was further classified into three categories (0, 1, and 2+) by taking account of its distribution and explainable meaning. Participants with a score of 0 were regarded as non-smartphone users, those with a score of 1 were regarded as traditional smartphone users because the majority of these were mainly used calling and messaging, and those with a score of 2+ were regarded as advanced users due to the more advanced functions they used. The NSFU indicates the acceptance and adoption of new technology.

Additionally, several demographic and socioeconomic characteristics (age, gender, region, marital status, education level, income, and occupation) as well as medical and health factors (smoking, drinking, hypertension, diabetes, and depression) were considered as covariates. These covariates were included to account for possible confounding effects since the literature suggested that they are closely related to the exposure (smartphone use) [28] or outcome (multi-domain cognition) [29]. Among these, smoking (or alcohol consumption) was measured by asking “Do you smoke cigarettes (drink alcohol)?” and three options of “never”, “smoking (drinking) now”, and “have quit now” were provided. Chronic diseases were assessed by the question “Do you suffer from the following physician-diagnosed chronic diseases (check all that apply)?” Hypertension and diabetes were included in this list of chronic diseases. In addition, depression was assessed by the 15-item Geriatric Depression Scale (GDS-15) and a GDS < 5 would be regarded as no depression [30].

### Statistical analysis

For the current study, 444 participants were excluded whose MoCA scores were very low (lower than the education-adjusted cut-offs for dementia, which were 11, 14, and 16 for ≤5, 6–8, and ≥ 9 years of education, respectively [31]) to avoid potentially occurring reverse causality since severe cognitive impairment likely impacts smartphone use. Additionally, 17 individuals with missing information with regard to smartphone use or smartphone function use were excluded. Finally, 2600 individuals were included in the study.

Firstly, demographic characteristics of the remaining 2600 participants were described via means and standard deviations or percentages. In particular, smartphone usage was summarized according to different functions stratified by gender, which were then assessed with the Chi-square test. Secondly, the characteristics of the adjusted MoCA total score and six subdomain scores were summarized according to gender specified NSFU (0, 1, and 2+) by using descriptive statistics (mean and standard deviations). The mean differences were compared via analysis of variance (ANOVA). Thirdly, general and subdomain cognitive functions were modelled on NSFU only, gender only, and NSFU and gender combined by using the seven proportional-odds cumulative logit model [32]. A four-category quantile classification was used for the total MoCA score (P_25_, P_50_, and P_75_) and this quantile and six subdomains’ score were used as the ordinal dependent variables for each model, respectively. All models were adjusted for the afore-mentioned covariates. For each model, a Chi-square score test indicated the appropriateness of the ordinal logistic regression; the parallel slopes assumption was not rejected at a *p*-value > 0.10. Since seven parallel analyses were performed, Bonferroni corrections were applied and thus, was set to 0.007. Fourthly, similar models were used to evaluate the joint associations of gender and NSFU on general and six subdomains of cognitive function by considering them based on the following possible mixed conditions: male & NSFU = 0, male & NSFU = 1, male & NSFU = 2, female & NSFU = 0, female & NSFU = 1, and female & NSFU = 2. Odds ratios (ORs) and corresponding 95% confidence intervals (CIs) were estimated, using men who did not report to have used smartphones as the reference category. All analyses were performed using SAS version 9.4 (SAS Institute, Cary NC, 2017).

## Results

### Characteristics of study participants

Descriptive statistics are presented in Table 1. Among the 2600 remaining participants, the mean age was 69.06 ± 7.06 years (ranging from 60 to 96 years). Male participants, who were currently married and lived in urban areas accounted for 53.88, 74.55, 54.62%, respectively. More than a quarter (25.33%) and almost two-thirds (64.51%) of respondents were illiterate and engaged in manual labor, respectively, while ~ 16% of older adults had insufficient income. Most respondents never smoked (62.38%) or drank alcohol (79.49%). More than one-third (35.04%) of the older adults suffered from hypertension while the prevalence of diabetes and depression were both ~ 10%. Overall, the education-adjusted mean MoCA score was 21.98 ± 4.61, and people who were younger, male, married, lived in urban areas, had higher education levels, had a balanced income, and had non-manual jobs were more likely to achieve higher MoCA scores.

**Table 1:** Characteristics of 2600 participants according to MoCA scores

	*N*(%)	MoCA score^a^
Overall		21.98 ± 4.61
Age (yr)		
60~	1558 (59.92)	22.69 ± 4.39
70~	779 (29.96)	21.55 ± 4.72
80~	263 (10.12)	19.01 ± 4.27
Gender		
men	1401 (53.88)	22.79 ± 4.31
women	1199 (46.12)	21.03 ± 4.78
Marital status		
unmarried	630 (25.45)	20.35 ± 4.56
married	1845 (74.55)	22.58 ± 4.49
Region		
rural	1180 (45.38)	20.77 ± 4.47
urban	1420 (54.62)	22.98 ± 4.49
Occupation		
manual	1674 (64.51)	20.99 ± 4.57
non-manual	921 (35.49)	23.76 ± 4.13
Income		
income < expenditure	408 (15.81)	20.62 ± 4.67
income = expenditure	1797 (69.62)	22.07 ± 4.53
income > expenditure	376 (14.57)	23.03 ± 4.59
Education level		
illiterate	655 (25.22)	17.98 ± 3.84
primary	933 (35.93)	22.06 ± 4.18
secondary and above	1009 (38.85)	24.49 ± 3.52
Smoking		
never smoke	1602 (62.38)	21.66 ± 4.73
previous smoke	177 (6.89)	22.76 ± 4.15
current smoke	789 (30.72)	22.46 ± 4.45
Drinking		
never drink	2050 (79.49)	21.73 ± 4.65
previous drink	106 (4.11)	22.43 ± 4.60
current drink	423 (16.40)	23.11 ± 4.30
Hypertension		
no	1665 (64.96)	21.95 ± 4.58
yes	898 (35.04)	22.05 ± 4.68
Diabetes		
no	2271 (89.34)	21.97 ± 4.63
yes	271 (10.66)	22.06 ± 4.57
Depression		
no	2273 (90.13)	22.26 ± 4.57
yes	249 (9.87)	19.74 ± 4.43

Note: The MoCA score^a^ has been adjusted according to the years of education

### Gender-related differences in characteristics of smartphone use

**Fig. 1:**
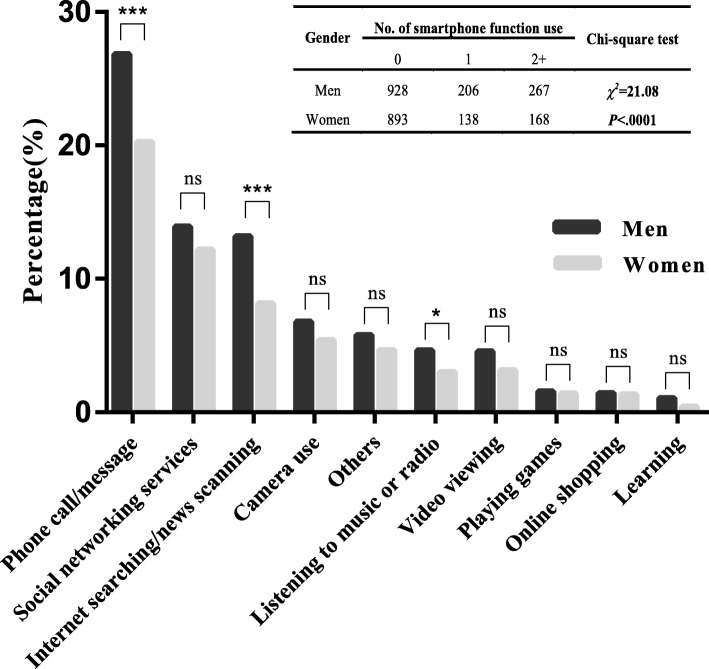
Gender differences in the characteristics of smartphone use. ^*^ denotes *p-value* < 0.05, ^***^ denotes *p-value* < 0.001, ns means no significant difference between groups

Figure 1 shows the gender differences with regard to the characteristics of smartphone use. Nearly 30% of the sampled older adults were smartphone users, 473 (60.72%) of which were men. The top three most frequently used smartphone functions were phone call/message (men: 26.84%, women: 20.27%), social networking services (men: 13.92%, women: 12.18%), and Internet searching/news reading (men: 13.20%, women: 8.17%). More men than women used each smartphone function with significant differences in phone call/message (*p* < 0.001), Internet searching/news scanning (*p* < 0.001), and listening to music or radio (*p* < 0.05).

### Characteristics of smartphone use according to the MoCA subdomain scores and ANOVA results

ANOVA (Table 2) indicated that, for both men and women, the mean scores of each cognitive domain differed significantly among groups with different NSFU, and higher scores in all subdomains were more likely to be attained by smartphone users with a NSFU of 2 + .

**Table 2:** Gender specified characteristics of 2600 participants according to the MoCA scores and ANOVA results

NSFU	*N*(%)	MoCA score^a^	Sub-score of MoCA in six cognitive domains (mean ± sd)					
			Memory	Visuospatial	Executive	Attention	Language	Orientation
Men								
0	928 (66.24)	21.84 ± 4.37	2.33 ± 1.91	2.17 ± 1.42	1.84 ± 1.10	5.04 ± 1.21	4.48 ± 1.31	5.69 ± 0.74
1	206 (14.70)	23.87 ± 3.56	3.08 ± 1.78	2.68 ± 1.19	2.18 ± 0.99	5.35 ± 0.92	4.91 ± 1.15	5.80 ± 0.58
2+	267 (19.06)	25.29 ± 3.35	3.12 ± 1.64	3.15 ± 1.03	2.73 ± 1.08	5.68 ± 0.63	5.20 ± 1.05	5.85 ± 0.42
*F*-value		82.55^*^	24.21^*^	57.58^*^	69.14^*^	38.59^*^	37.07^*^	7.35^*^
Women								
0	893 (74.48)	20.12 ± 4.59	2.61 ± 1.89	1.36 ± 1.38	1.47 ± 1.02	4.52 ± 1.46	4.00 ± 1.44	5.65 ± 0.69
1	138 (11.51)	22.38 ± 4.66	2.96 ± 1.90	2.04 ± 1.37	1.98 ± 1.14	5.03 ± 1.13	4.62 ± 1.34	5.75 ± 0.69
2+	168 (14.01)	24.75 ± 3.68	3.18 ± 1.70	2.71 ± 1.25	2.41 ± 1.11	5.49 ± 0.93	5.10 ± 1.11	5.87 ± 0.34
*F*-value		82.64^*^	6.99^*^	74.2^*^	60.27^*^	39.26^*^	49.17^*^	8.23^*^

Note: ^a^ The MoCA score has been adjusted according to the years of education; ^*^ denotes *p* < 0.007; NSFU represents the number of smartphone functions used

### Associations of NSFU and gender with six subdomains and general cognition

NSFU was positively associated with general cognition and the cognition of all other subdomains except for memory and orientation (model 1 in Table 3), and increasing associations were observed between them with higher NSFU level except for memory and orientation. With or without constant NSFU, significant differences were not found in general cognition between men and women; however, women significantly outperformed men in memory while being surpassed by men in visuospatial ability (models 2 and 3).

**Table 3:** ORs (95% CIs) obtained from modelling general and sub-domains cognition on NSFU and gender

		MoCA score^a^	Sub-score of MoCA in six cognitive domains (mean ± sd)					
			Memory	Visuospatial	Executive	Attention	Language	Orientation
Model 1^b^								
NSFU	0	1.00	1.00	1.00	1.00	1.00	1.00	1.00
	1	**1.41 (1.11–1.78)** ^*^	1.29 (1.01–1.65)	1.31 (1.04–1.66)	**1.48 (1.17–1.88)** ^*^	1.10 (0.86–1.41)	**1.52 (1.20–1.93)** ^*^	1.29 (0.91–1.83)
	2+	**2.22 (1.75–2.82)** ^*^	1.16 (0.93–1.46)	**1.98 (1.57–2.50)** ^*^	**2.37 (1.88–2.98)** ^*^	**1.78 (1.36–2.34)** ^*^	**1.94 (1.53–2.46)** ^*^	1.25 (0.87–1.80)
Model 2^c^								
Gender	men	1.00	1.00	1.00	1.00	1.00	1.00	1.00
	women	0.86 (0.69–1.07)	**1.38 (1.10–1.73)** ^*^	**0.46 (0.37–0.57)** ^*^	0.82 (0.66–1.03)	0.79 (0.63–1.00)	0.91 (0.73–1.13)	0.95 (0.69–1.30)
Model 3^d^								
NSFU	0	1.00	1.00	1.00	1.00	1.00	1.00	1.00
	1	**1.40 (1.11–1.77)** ^*^	1.31 (1.02–1.67)	1.28 (1.01–1.62)	**1.47 (1.16–1.87)** ^*^	1.09 (0.85–1.40)	**1.52 (1.20–1.93)** ^*^	1.29 (0.91–1.83)
	2+	**2.22 (1.75–2.81)** ^*^	1.18 (0.94–1.48)	**1.92 (1.52–2.43)** ^*^	**2.35 (1.87–2.96)** ^*^	**1.77 (1.35–2.32)** ^*^	**1.93 (1.53–2.45)** ^*^	1.25 (0.87–1.80)
Gender	men	1.00	1.00	1.00	1.00	1.00	1.00	1.00
	women	0.87 (0.7–1.09)	**1.39 (1.11–1.75)** ^*^	**0.47 (0.38–0.59)** ^*^	0.85 (0.68–1.06)	0.81 (0.64–1.02)	0.93 (0.75–1.16)	0.96 (0.70–1.31)

Note: All three models adjusted for the same set of covariates: age, region, marital status, level of education, income, occupation, smoking, drinking, hypertension, diabetes, and depression

^b^NSFU entered in the model in addition to the above covariates

^c^Gender entered in the model in addition to the above covariates

^d^Both NSFU and gender entered in the model in addition to the above covariates

^a^The MoCA score has been adjusted according to the years of education;

^*^denotes *p* < 0.007; NSFU represents the number of smartphone functions used

### Joint association of smartphone use and gender on general and multi-domain cognition

**Fig. 2:**
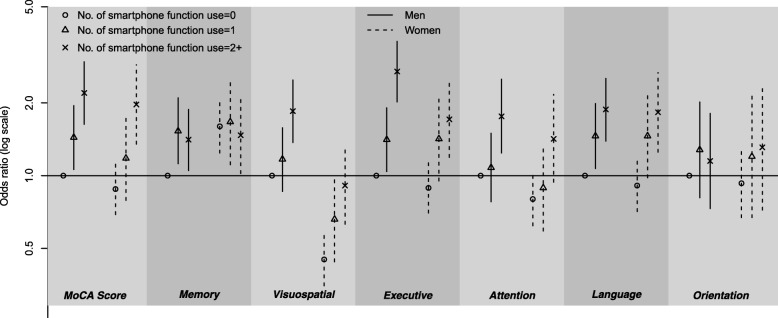
Joint associations of the number of smartphone functions used (NSFU) and gender with general cognition and six sub-domains. Proportional odds models were adjusted for background factors (age, region, marital status, education level, marital status, income, and occupation) as well as medical and health factors (smoking, drinking, hypertension, diabetes, and depression). Symbols represent the odds ratios (ORs) while vertical bars around the symbols indicate the corresponding 95% confidence intervals (CIs). Universal reference group: NSFU = 0 & gender = men

Figure 2 shows ORs with corresponding 95% CIs for joint associations of NSFU and gender on general and sub-domain cognitive function. Male with NSFU = 0 was used as the general reference group, and an increasing pattern in the odds of general and four domains of cognitive ability was observed with increasing NSFU level (except for memory and orientation). In general, men with a NSFU of 2+ had the highest odds of better cognition (OR (95% CI): 2.20 (1.63–2.96)). For non-smartphone users, women significantly performed better in memory than men (OR (95% CI): 1.60 (1.24–2.06)); however, this advantage might be tempered with higher NSFU. In contrast, with regard to their visuospatial ability, women performed significantly worse than men (OR (95% CI): 0.45 (0.35–0.58)) for non-smartphone users, and this disadvantage became insignificant for females with a NSFU of 2+. With regard to executive function, attention, language, and orientation, women had an inferior position at first compared to men, while they were better with a NSFU of 2+, with each OR = 1.71, 1.42, 1.83, and 1.31, respectively (although not all of these were significant).

## Discussion

Based on this large-scale survey, gender differences in smartphone function use were explored. Associations of NSFU and gender with multi-domain cognition were detected, and joint associations of gender and NSFU were further explored with regard to general and multi-domain cognitive functions. Smartphone use among older adults was comparatively low, especially in women, and a significant gender difference was found in phone call/message, Internet searching/news scanning, and listening to music or radio. Smartphone use was positively associated with multi-domain cognitive functions. Except for memory, women performed worse than men in all other cognitive sub-domains; however, luckily, this gender gap could be bridged at a NSFU of 2 + .

In general, older adults reported a low percentage of smartphone use (29.96%) while smartphones are a necessity in the daily life of young people [33] and mobile payments have become the mainstream in China [34]. Nevertheless, similar results were also reported for developed countries. For example, only 27% among US aged 65+ [35] and 31.91% of persons aged 50+ in Hong Kong [15] used smartphones. The comparatively low usage may be because smartphone use has developed rapidly in recent years along with an ever increasing number of functions. In such a fast changing context, older adults find it more difficult to learn how to use smartphones than younger people and experience frustration during the learning process, which may deter their further learning and usage [15, 36]. As expected, this study also showed that older adults tend to use smartphones more for socializing (phone call/social networking service) than for entertainment (listening to music/radio, viewing videos, or playing games), which is in accordance with the results of Chen et al. [37]. An age-friendly smartphone design, that is not only tailored to older adults’ age-related sensory-perceptual changes, such as using large characters, loud volume, and easy-to-operate menu, but also based on their user needs and requirements, is required to increase the number of elderly smartphone users.

Smartphone users had an advantage in multi-domain cognitive functions compared to their non-smartphone counterparts and the higher their NSFU, the more benefits they attained (except for memory and orientation). Similar benefits were also reported for both computer use [38] and Internet use [39]. This result is consistent with the mental-exercise hypothesis “Use it or lose it”, i.e., engaging more in mentally stimulating activities leads to better performance in cognition-related tasks [40, 41]. Furthermore, experimental data also indicates that new neurons are kept alive by effortful learning [42]. Concretely, smartphone users have to be familiar with the spatial layout of the number pad and various functional buttons in order to operate a smartphone, which may stimulate their visuospatial ability. Moreover, similar to TV, smartphones can serve as a real-time information provider with pictures or videos, which greatly attract viewers [43] and which might be beneficial for their attention. In addition, language skills of smartphone users may be enhanced by phoning or using social media [44]. Besides, the executive function can benefit from the process of using any basic or advanced applications [45]. No association was found between NSFU and orientation, which may be because bi-dimensional views may not be useful for spatial orientation ability [46] despite the fact that smartphones can serve as visual stimulus for its users.

Women outperformed men in memory-related tasks but were surpassed by men in visuospatial function, which is consistent with previous studies [20, 21, 47]. While men’s memory ability was significantly higher in individuals who used a smartphone, no positive gradient with higher NSFU was observed. Furthermore, female smartphone users with NSFU of 2+ even almost lost their originally significant advantage in memory. This suggests that over dependence on smartphones may negatively impact the users’ memory as it may decrease opportunities for users to remember things without the help of a smartphone. Moreover, although women were in an inferior position in visuospatial ability compared to men, joint association results indicated that this inferiority could be overcome when females had a NSFU of 2+. A similar result was reported by Feng et al. [22]. In addition, compared to men who did not use smartphones, female users with NSFU of 2+ also presented better general cognition, language, and executive ability, indicating the potentiality of smartphone use in bridging gender differences in cognitive functions. However, this potentiality was not significant when female users only used one function despite less women reporting 2+ NSFU (14.01%) than men (19.06%) in the current study. This may result from technophobia reported among older female users [48]. Luckily, the acceptance of technology by senior female users can be encouraged by their family members [49]. Hence, to help with women’s cognitive enhancement, they are encouraged to learn more smartphone functions.

### Strengths and limitations

This study has a number of strengths. One of the main strengths is that it focused on the association of using smartphones with cognitive function, which has rarely been studied before. Moreover, six domain-specific cognitive functions were simultaneously considered by gender, and furthermore, a joint association between gender and smartphone use on multi-domain cognition was detected, which offers a new and more comprehensive perspective for specific individual interventions. In addition to, the conducted survey was based on a large randomly selected sample, which allows for greater generalization of the obtained results. Nonetheless, the current study has several limitations. First, this study is a cross-sectional study, and no causal effect could be concluded between smartphone use and multi-domain cognition. However, to decrease the reverse causality to a certain extent, participants with dementia or with severe cognitive impairment were excluded since demented subjects will have greater limitations with respect to smartphone use. The remaining participants, including cognitively normal participants and those with mild cognitive impairment, should have normal ability with regard to daily activities [50]. Second, the associations of the domain-specific cognition with the number of smartphone function use were explored; however, associations with specific smartphone functions are still missing. However, we will focus on this next and will strive to identify the most beneficial smartphone functions corresponding to each domain-specific cognition. Third, no detailed information on the habit of smartphone use was collected, such as frequency and length of usage time, which obstructs a deeper understanding of its relationship with cognition. Fourth, domain-specific cognitive functions were assessed by the MoCA only, which might impede a stable result. Moreover, although only the MoCA was used as a cognitive screener at first, a host of studies demonstrated its stability for cognitive assessment [51, 52]. Nevertheless, more fine-grained neuropsychological assessment methods should be used to gain a more stable result and to achieve better comparability to similar studies.

## Conclusion

This study detected the joint association of smartphone use and gender on multi-domain cognitive functions. In conclusion, smartphone users had an advantage in multi-domain cognitive functions compared to non-smartphone users and the more smartphone functions they engaged in, the more benefits they attained. Furthermore, gender differences were found in visuospatial ability and memory, which could be overcome by smartphone use.

## Abbreviations

CIs: Confidence Intervals
GDS: Geriatric Depression Scale
MoCA: Montreal Cognitive Assessment
NSFU: the number of smartphone functions used
ORs: Odds Ratios
